# Coronavirus Disease (COVID-19) and the risk of Post-Traumatic Stress Disorder: A mental health concern in Nepal

**DOI:** 10.3126/nje.v10i2.29761

**Published:** 2020-06-30

**Authors:** Mohammad Asim, Edwin van Teijlingen, Brijesh Sathian

**Affiliations:** 1 Surgery Department, Trauma Surgery, Hamad General Hospital, Doha, Qatar; 2 Centre for Midwifery, Maternal and Perinatal Health, Bournemouth University, Bournemouth, UK; 3 Manmohan Memorial Institute of Health Sciences, Nepal; 4 Nobel College, Nepal; 5 Geriatric Medicine Department, Rumailah Hospital, Hamad Medical Corporation, Doha, Qatar

## Background

The sudden outbreak of Coronavirus-19 disease (COVID-19) is transforming the psychology and interpersonal relationships of millions across the globe. Sense of fear and uncertainty are the initial emotional response to a pandemic which can be overwhelming and gradually move towards negative emotions such as stress, anxiety and depression that can lead to social discontent and mental health illness [1]. The negative socio-economic consequences and traumatic experience of COVID-19 may aggravate the underlying mental health disorders or serve as a precursor for new psychiatric illness, this is true for COVID-19 patients, relatives, health care workers and those not suffering from COVID-19, but are affected by the lockdown imposed by governments to reduce the spread of the infection. Currently, the COVID-19 infection and its associated mortality are still high in many countries which are negatively impacting the psychological well-being of individuals and societies.

Nepal is one of the Lower Middle-Income Countries (LMICs) sharing borders with India and China. Like many South Asian countries, the COVID-19 outbreak greatly impacted the healthcare system in Nepal and particularly mental health services are affected due to minimal resources (funding & staff) and gaps in treatment [2,3]. Until 25 June 2020, there were 11,162 COVID-19 cases in Nepal most were young (21-30 years followed by 31-40 years) which is the most productive age group [4].

### COVID-19 and Post-Traumatic Stress Disorder

Posttraumatic stress disorder (PTSD) is a severe mental illness that may develop in individuals who have experienced or witnessed a traumatic event [5]. It can negatively affect the functional and social wellbeing of patients and PTSD has four core features: (a) re-experiencing the traumatic event; (b) avoidance of situations; (c) experiencing negative changes in emotions and beliefs; and (d) hyperarousal [6]. However, these symptoms may develop immediately after one month or may appear after few months or even years after exposure [7]. Interestingly, not all individuals who are exposed to a traumatic events or stressful situation develop PTSD. The risk of developing PTSD depends on the individual’s initial response to the traumatic event, the intensity of the memory, coping style, feeling of safety and social support.

### Contemporary literature on COVID-19 and PTSD

Over the past decades, PTSD has been recognized as a potential mental health disorder. As, COVID-19 is a novel emergency situation, there are only a few pioneering studies on its effects on mental health. Studies from China reported that COVID-19 can cause traumatic experiences to the patients and caregivers which may lead to PTSD and/or psychological disorders [8-9]. It has been estimated that around 20-25% of such patients may develop PTSD symptoms. Bo et al. [10] assessed 714 COVID-19 patients online before discharge from quarantine and reported posttraumatic stress symptoms in nearly all (96.2%). The authors suggested that the higher rate of symptoms were partly attributed to the hospitals quarantine which last for weeks preventing social contact with family and friends. Whilst, Liu et al. [8] reported lower prevalence of COVID-19-related PTSD symptoms (7%) among 285 adult patients from Wuhan, China. Sun et al. [9] found a similarly low prevalence of posttraumatic stress symptoms (4.6%) amongst 2091 participants. An Italian web-based cross-sectional survey for COVID-19 also identified a higher prevalence of PTSD symptoms (29.5%) [11]. A cross-sectional study of 950 civilians quarantined for COVID-19 suggested that the quarantine resulted in psychological distress and PTSD which persisted even after quarantine [12]. A recent meta-analysis on psychiatric presentations of patients with severe coronavirus infections showed point prevalence of PTSD, depression and anxiety disorders to be 32.2%, 14.9% and 14.8%, respectively [13]. Results of the previous outbreaks highlighted that post infection many patients may develop mental illnesses and some may end-up with PTSD. However, in comparison to the earlier outbreaks of coronavirus infections, the COVID-19 pandemic has a higher frequency of hospitalisation and mortality worldwide. Moreover, the risk factors for psychological illness associated with COVID-19 are not yet determined fully.

### Predisposing factors for PTSD among COVID-19 survivors

To date, there is limited information available on the psychological impact of COVID-19 on patients, caregivers and healthcare workers. Hospitalisation due life-threatening COVID-19 illness appeared to be a risk factor for developing PTSD [9-10], as is being quarantined [14]. Notably, patients with COVID-19 are more likely to experience stigmatisation and rejection which can also increases the risk of PTSD [15]. Another risk factor for PTSD is the death of loved ones secondary to COVID-19 infection. Also, poorer people are more likely to experience stressful situations enhancing the risk of PTSD. Studies showed that women are more vulnerable to PTSD than men [8-9]. Jiang et al. [16] recent showed that older individuals in China perceived less posttraumatic stress as compared to young individuals post COVID-19.

### Healthcare workers and risk of PTSD

Recent studies have demonstrated that frontline healthcare professionals managing COVID-19 patients are at increased risks of developing mental health issues such as stress, insomnia, depression, and anxiety [17-19]. Health professionals working in COVID-19 quarantine units with lack of proper protective measures and those witnessed the death of fellow doctors are more likely to develop symptoms of PTSD [20]. This editorial argues that particular attention should be focused on healthcare workers in high-risk settings such as hospitals, quarantine facilities, nursing homes and long-term care facilities. Therefore, it is crucial to identify the major risk factors for PTSD in frontline healthcare workers.

### Preparedness and management of PTSD

One solution to address mental health problems is to provide mental health services at primary care centres and satellite clinics [21]. It is crucial to screen COVID-19 patients for symptoms of PTSD/mental illness. However, the general reluctance to visit the health care centres during the pandemic is a major concern. Also, mental health professionals should focus on the underlying PTSD risk factors among survivors of COVID-19 and provide evidence-based therapeutic interventions. The PTSD treatment mainly includes psychotherapies such as cognitive behavioural therapy (CBT) and eye movement desensitisation and reprocessing (EMDR). Also, there are potential pharmacotherapies, but drug treatments for PTSD should not be used as routine first-line treatment in adults [7]. Moreover, it is important to develop coping strategies with the help of alternative treatments such as yoga, meditation and acupuncture.

During a pandemic, it may be difficult for patients to get a psychiatric consultation; so telepsychiatry or telemedicine to contact patients for assessment via the internet can be a cost-effective solution [22]. In case of unavailability of the internet, a local mental health professional or a pharmacist could use a telephonic conversation or short message service (SMS). However, currently telepsychiatry is not a part of routine patient care, nor is there a standard consensus guideline for its implementation. Therefore, it is important that Nepal Medical Council (NMC) in collaboration with the Ministry of Health and Population (MoHP) in Nepal should initiate a free helpline service to deal with various mental illnesses. This telephone service should be toll free available daily from 8am to 8pm, managed by a team of mental health experts to provide assessment and instant support. Special focus should be given to high-risk population such as young adults, children and their parents, elderly and frontline health workers.

## Conclusions

In Nepal, there is a need for national mental health surveys post COVID-19. This pandemic can cause traumatic experiences to the patients, caregivers, those quarantined and frontline healthcare providers which may lead to PTSD. Moreover, determining the major risk factors for PTSD and other mental illnesses is essential to help reduce the mental health burden of COVID-19. Special attention should be focused on high-risk individuals, including policies to implement regular screening of PTSD symptoms. Currently, telepsychiatry is not a part of routine patient care therefore, it is important that a free telephone helpline service should be established by the NMC and MoHP to deal with various mental health problems caused or exacerbated by the current pandemic.
